# Bone Union Assessment with Computed Tomography (CT) and Statistical Associations with Mechanical or Histological Testing: A Systematic Review of Animal Studies

**DOI:** 10.1007/s00223-021-00904-6

**Published:** 2021-08-21

**Authors:** A. Willems, C. Iҫli, J. H. Waarsing, S. M. A. Bierma-Zeinstra, D. E. Meuffels

**Affiliations:** 1 Department of Orthopaedics and Sports Medicine, Erasmus MC University Medical Centre, Doctor Molewaterplein 40, 3015GD Rotterdam, The Netherlands; 2 Department of General Practice, Erasmus MC University Medical Centre, Rotterdam, The Netherlands

**Keywords:** Bone union, Computed tomography, Torsional test, Three-point bending test, Histological test, Torsional rigidity

## Abstract

Objective and accurate assessment of bone union after a fracture, arthrodesis, or osteotomy is relevant for scientific and clinical purposes. Bone union is most accurately imaged with computed tomography (CT), but no consensus exists about objective assessment of bone union from CT images. It is unclear which CT-generated parameters are most suitable for bone union assessment. The aim of this review of animal studies is to find which CT-generated parameters are associated most strongly with actual bone union. Scientific databases were systematically searched. Eligible studies were studies that (1) were animal studies, (2) created a fracture, (3) assessed bone union with CT, (4) performed mechanical or histological testing as measure of actual bone union, and (5) associated CT-generated outcomes to mechanical or histological testing results. Two authors selected eligible studies and performed risk of bias assessment with QUADAS-2 tool. From 2567 studies that were screened, thirteen studies were included. Most common CT parameters that were investigated were bone mineral density, bone volume, and total callus volume. Studies showed conflicting results concerning the associations of these parameters with actual bone union. CT-assessed torsional rigidity (assessed by three studies) and callus density (assessed by two studies) showed best results. The studies investigating these two parameters reported moderate to strong associations with actual bone union. CT-assessed torsional rigidity and callus density seem the most promising parameters to represent actual bone union after a fracture, arthrodesis, or osteotomy.

*Prospero trial registration number*: CRD42020164733

## Introduction

Achieving bone union is the main goal in patients after a fracture, osteotomy, or arthrodesis. But when has bone healed? This is a simple question, but the answer is rather complicated.

In the clinic, bone union is generally assessed based on conventional radiographs and on clinical examination, such as response to weight bearing or palpation of the fracture [1]. However, assessing bone union is a rather subjective decision [2], and the lack of consensus has been extensively described by several studies [3, 4].

Assessment of bone union after a fracture, arthrodesis, or osteotomy is an important clinical consideration. Wrong assessment of bone healing can have major negative consequences for a patient. By overestimating the amount of bone healing, a bone might be loaded too early resulting in a displaced fracture or failure of osteosynthesis material. Underestimating bone healing may cause unnecessary immobilization resulting in stiffness, decreased muscle mass and function, and productivity loss of the patient [5, 6]. Especially if bone union is doubtful, an objective and accurate assessment tool can be helpful in clinical decision-making. Also, for scientific purposes, an objective and accurate method of fusion assessment would be of high value. Being able to accurately assess bone union would have several advantages like a decreased risk of biases within studies and less patients needed in clinical trials with bone union as primary outcome. Additionally, it would become easier to compare results between studies. In orthopedic studies, bone union is a commonly used primary outcome, for instance in studies investigating bone healing stimulating therapies after a fracture, osteotomy, or arthrodesis [7–9]. For the objective assessment of bone healing from radiographs, the radiographic union score (RUS) has been introduced in 2012 [10, 11]. Ever since, this semi-quantitative assessment tool for assessment of fracture healing has become increasingly popular as an outcome measure in clinical studies [12, 13]. However, computed tomography (CT) is the best method to image bone and has been shown to be superior to plain radiographs, MRI, and DEXA to assess bone union [14–17]. For CT, no golden standard exists for the objective assessment of bone union as an outcome measure. Therefore, we would like to create a method to objectively assess bone union from CT. This could then be used as golden standard for bone union assessment in clinical studies, but could also be used in the clinic if bone fusion after fracture, arthrodesis, or osteotomy is doubtful.

To establish an objective clinically applicable tool for bone union assessment, we need to know which CT-generated outcomes have a strong association with actual bone union. This review will therefore investigate which CT parameters are associated with actual bone healing. Actual bone union will be tested by mechanical or histological tests. As it is unethical and therefore impossible to acquire this data in clinical studies, in this review, we focus on animal studies. The aim of this review is to find CT parameters that best represent actual bone union, which is indicated by mechanical or histological testing.

## Method

The protocol of this review has been prospectively registered at the International prospective register of systematic reviews (http://www.crd.york.ac.uk/prospero/; registration number CRD42020164733).

To find all studies concerning the assessment of bone union with CT, an online search was performed on February 5, 2020. Five online databases were searched (Embase.com, Medline Ovid, Web of science, Cochrane CENTRAL, and Google Scholar). The search strategy for Medline Ovid is presented in Table 1 and was adapted for the other databases. Following the selection of eligible articles, reference lists of eligible articles were checked for missed articles.

**Table 1:** Search strategy for Medline Ovid

(fracture healing/ OR Fractures, Ununited/ OR (((bone* OR fracture* OR arthrodes* OR osteotom* OR scaphoid* OR osseous OR bony) ADJ6 (healing OR union* OR nonunion* OR united OR ununited OR consolidation))).ab,ti.) AND (exp "Validation Studies"/ OR "Comparative Study"/ OR exp "psychometrics"/ OR "outcome assessment (health care)"/ OR exp "observer variation"/ OR exp "Health Status Indicators"/ OR exp "reproducibility of results"/ OR exp "discriminant analysis"/ OR (psychometr* OR clinimetr* OR clinometr* OR (outcome ADJ3 (assessment* OR measure*)) OR (observer* ADJ3 variation*) OR ((reproducib* OR reliab* OR unreliab* OR valid* OR coefficient OR homogeneity OR homogeneous OR generaliza* OR generalisa* OR concordance OR repeatab* OR discriminative OR known group OR subscale* OR sensitiv* OR responsive* OR error OR errors) ADJ6 (diagnos* OR observ* OR tomograph* OR radiodiagnos* OR radiograph* OR x-ray*)) OR ((dimension*) ADJ6 (diagnos* OR observ* OR tomograph* OR radiodiagnos* OR radiograph* OR x-ray*) NOT (3-dimension* OR three-dimension*)) OR (internal* ADJ3 consisten*) OR (cronbach* ADJ3 (alpha OR alphas)) OR (item ADJ3 (correlation* OR selection* OR reduction*)) OR agreement OR precision OR imprecision OR (precise* ADJ3 value*) OR (test ADJ3 retest) OR (reliab* ADJ3 (test OR retest)) OR interrater* OR inter-rater* OR intrarater* OR intra-rater* OR intertester* OR inter-tester* OR intratester* OR intra-tester* OR interobserver* OR inter-observer* OR intraobserver* OR intra-observer* OR intertechnician* OR inter-technician* OR intratechnician* OR intra-technician* OR interexaminer* OR inter-examiner* OR intraexaminer* OR intra-examiner* OR interassay* OR inter-assay* OR intraassay* OR intra-assay* OR interindividual* OR inter-individual* OR intraindividual* OR intra-individual* OR interparticipant* OR inter-participant* OR intraparticipant* OR intra-participant* OR kappa OR kappa-s OR kappas OR ((replicab* OR repeated) ADJ3 (measure OR measures OR findings OR result OR results OR test OR tests)) OR (intraclass ADJ3 correlation*) OR (factor ADJ (analys* OR structure*)) OR (multitrait ADJ3 scaling ADJ3 (analysis OR analyses)) OR item discriminant OR (interscale ADJ3 correlation*) OR ((individual OR interval OR rate OR analysis OR values) ADJ3 variabil*) OR (uncertainty ADJ3 (measurement OR measuring)) OR standard error of measurement OR (limit ADJ3 detection) OR minimal detectable concentration OR interpretab* OR ((minimal OR minimally OR clinical OR clinically) ADJ3 (important OR significant OR detectable) ADJ3 (change OR difference)) OR (small* ADJ3 (real OR detectable) ADJ3 (change OR difference)) OR meaningful change OR ceiling effect OR floor effect OR Item response model OR Rasch OR Differential item functioning OR computer adaptive testing OR item bank OR cross-cultural equivalence OR ((defin* OR assess*) ADJ3 quanti*) OR (classif* ADJ3 (union OR consolidat*)) OR (union ADJ3 Score*)).ab,ti.) AND (exp Tomography, X-Ray Computed/ OR exp radiography/ OR Arthrography/ OR Diagnostic Imaging/ OR X ray film/ OR exp radiologists/ OR ((compute* ADJ3 tomograph*) OR radiodiagnos* OR radiolog* OR radiograph* OR x-ray* OR ct OR (cat ADJ (scan*)) OR rontgen* OR roentgen* OR microCT OR ((bone* OR diagnos*) ADJ3 imaging)).ab,ti.) NOT (letter OR news OR comment OR editorial OR congresses OR abstracts).pt. NOT (case reports/ OR case report.ti.)	

After the search of the databases, eligible articles were selected, by two authors (AW and CI), based on predefined eligibility criteria (Table 2). Overall, we included studies that created a fracture in the appendicular skeleton of an animal. A fracture was defined as a bone gap that was created by performing an osteotomy or by impact loading. Studies with distraction osteogenesis or bony defects were excluded. Bony defects were defined as drilling a hole in a bone. After at least 4 weeks, CT should be performed to assess bone union. The time period of 4 weeks was chosen because we aim to look at more advanced fracture healing and are not interested in the very early stages of bone healing. Simultaneously with CT, actual bone union should be tested by mechanical or histological testing. Parameters that are obtained from mechanical or histological testing and reflect bone union could be, for instance, bone mineral density, bone volume, or cross-sectional area. The association between CT outcomes and mechanical or histological outcomes should thereafter be statistically examined.

**Table 2:** In- and ex-clusion criteria

Inclusion	Exclusion
• Animal study	• Bony defects or Distraction osteogenesis
• Bone fracture of the appendicular skeleton	
• Aim of the study to quantify bony union with micro CT, quantitative CT, multidetector CT, cone beam CT, or clinical CT	• Follow-up period < 4 weeks
	• Data have been published before
• The relation between CT and histological or mechanical testing is statistically assessed	• Review article
	• Full text not available
• Article in English, Spanish, German, or Dutch	

Firstly, based on the predefined in- and ex-clusion criteria, the eligibility of studies was assessed by reading title and abstract. Secondly, both authors read the full text of the pre-selected studies and assessed eligibility. After the first and second round, the study selection of both authors was compared. In case of disagreements, a third reviewer decided (DM).

Data were extracted from eligible studies using a predefined data extraction sheet. Data extraction was performed by one reviewer (AW) and checked by a second reviewer (CI). Disagreements were resolved by reaching consensus. Data that were extracted from the studies were data related to the methodology of the studies (fracture site, number of animals, animal species, use of bone growth stimulating injections, time till CT, type of CT, CT settings, volume of interest, threshold for bone, performance of histological testing and mechanical testing, mechanical test that was performed), outcome measures (outcomes of mechanical or histological testing, and outcomes of CT), and statistical associations between CT-outcomes and mechanical or histological testing.

Risk of bias assessment was done with the QUADAS-2 tool [18], which is a tool for diagnostic studies. Although the tool was originally designed for human studies, we chose this tool because it is the best available tool to assess risk of bias for studies in this review. The risk of bias assessment was done by two authors (AW and CI), and discrepancies were resolved by reaching consensus.

The primary outcome of this systematic review will be the strength of the associations between CT-assessed outcomes and mechanical or histological tested bone union. These associations can be expressed as Pearson’s correlation coefficients, coefficients of determination, or strength of association in a regression model. To improve readability of this review, all linear Pearson’s correlation coefficients will be squared, resulting in coefficients of determination. To distinguish between weak and strong relations, coefficients of determination will be classified as weak (R^2^ < 0.4), moderate (R^2^ = 0.4–0.7), and strong (R^2^ > 0.7).

## Results

**Fig. 1:**
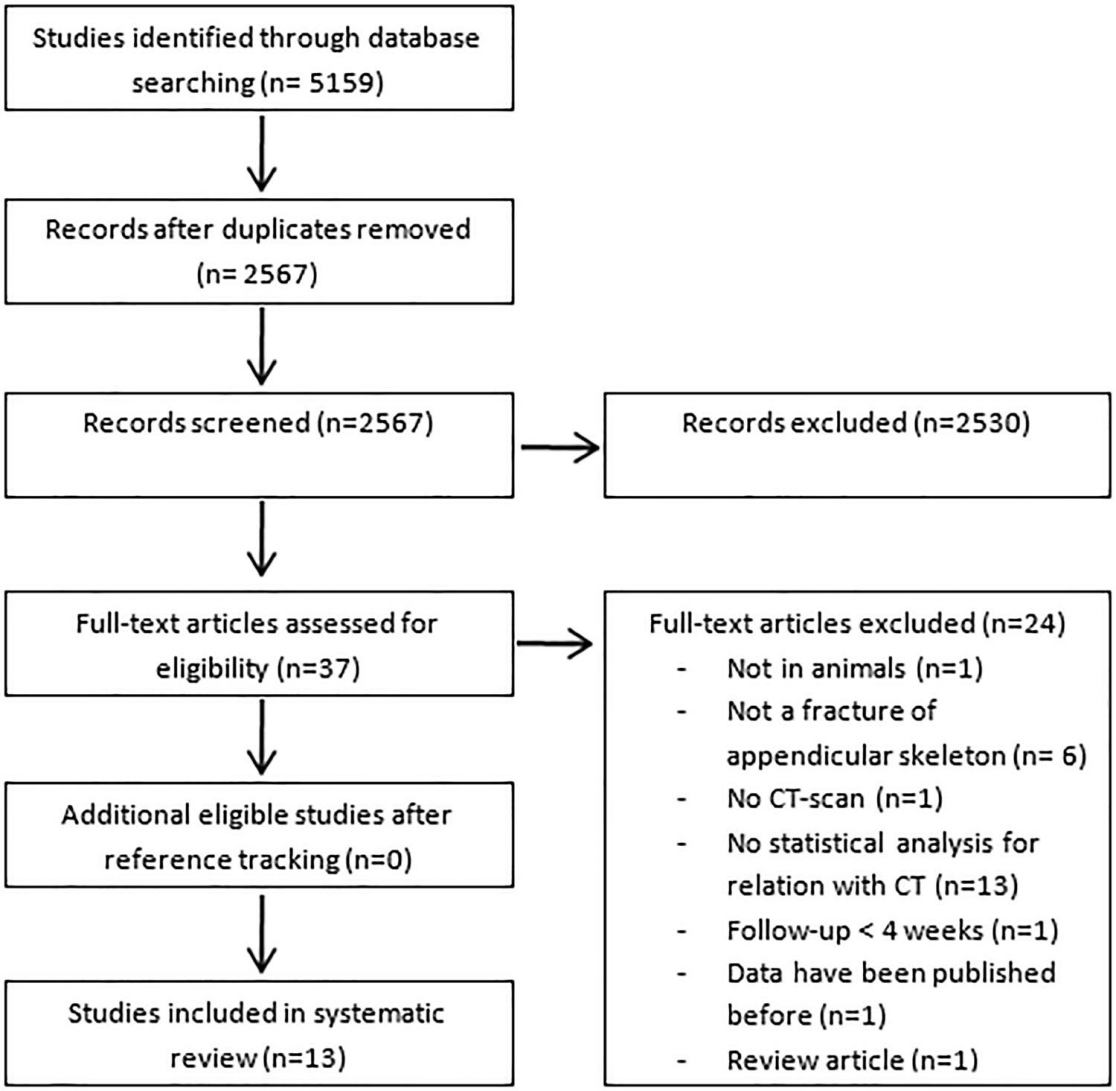
Flow chart of study selection

The search initially resulted in 5159 studies. After removing the duplicates, 2567 studies were screened on title and abstract, resulting in 38 potentially eligible studies. After reading the full-text of those studies, thirteen studies were included in our systematic review (Fig. 1).

The results of the risk of bias assessment with the QUADAS-2 tool are presented in Table 3. The assessment showed that risk of bias is generally low in the domains ‘animal selection’ and ‘flow and timing.’ However, twelve studies did not clearly describe whether results of the index test (CT) were interpreted without the knowledge of the results of the reference test (mechanical or histological testing) and vice versa. Therefore, the risk of bias concerning these domains is unclear.

**Table 3:** Risk of bias assessment with the QUADAS-2 tool

Study	Risk of bias				Applicability concerns		
	Patient selection	Index test	Reference standard	Flow and timing	Patient selection	Index test	Reference standard
Mehta (2013) [29]	☺	☺	☺	☺	☺	☺	☺
Morgan (2009) [27]	☺	?	?	☺	☺	☺	☺
Nyman (2009) [26]	☺	?	?	☺	☺	☺	☺
Shefelbine (2005) [30]	☺	?	?	☺	☺	☺	☺
Nazarian (2010) [19]	☺	?	?	☺	☺	☺	☺
Fiset (2018) [22]	☺	?	?	☺	☺	☺	☺
Jämsä (2000) [21]	☺	?	?	☺	☺	☺	☺
Sigurdsen (2011) [25]	☺	?	?	☺	☺	☺	☺
Markel (1990) [17]	☺	?	?	☺	☺	☺	☺
Augat (1997) [20]	☺	?	?	☺	☺	☺	☺
Den Boer (1998) [28]	☺	?	?	☺	☺	☺	☺
Wright (2012) [24]	☺	?	?	☺	☺	☺	☺
Böhm (1999) [23]	☺	☹	?	☺	☺	☺	☺

☺Low risk, ☹High risk, ? Unclear risk

### General Study Characteristics

The studies created a fracture by performing an osteotomy (eight times) [17, 19–25] or by impact loading (five times) [26–30]. Six studies created the fracture in the femur [19, 22, 26, 27, 29, 30], six in the tibia [17, 20, 21, 24, 25, 28], and one in the metatarsus [23]. During follow-up, eight studies used micro-CT for the assessment of fracture healing [19, 22, 24–27, 29, 30], two studies peripheral quantitative CT [20, 21], and three studies (quantitative) clinical CT [17, 23, 28]. All studies performed mechanical testing, such as torsional tests [17, 19, 22, 24, 27–30], three-point bending tests [20, 23, 26], or axial tests [17, 21]. Two studies also performed histological testing [20], but one of those did not correlate the outcomes to CT outcomes [17]. See Table 4 for animal species that were used and more study characteristics.

**Table 4:** General study characteristics

Study	Fractured bone	Number of animals	Animal species	Bone-stimulating injection	Time till CT (weeks)	Type of CT	Histological testing	Mechanical testing
Mehta (2013) [29]	Femur	99	Mice	No	2–5	Micro	No	Torsional Testing
Morgan (2009) [27]	Femur	72	Mice	Yes	2–7	Micro	No	Torsional testing
Nyman (2009) [26]	Femur	53	Rats	Yes	4	Micro	No	Destructive three-point bending test
Shefelbine (2005) [30]	Femur	50	Rats	No	3, 4	Micro	No	Torsional Testing
Nazarian (2010) [19]	Femur	10	Rats	Yes	8	Micro	No	Torsional testing
Fiset (2018) [22]	Femur	29	Rats	No	5–9 or 17	Micro	No	Torsional testing
Jämsä (2000) [21]	Tibia	141	Rats	No	4 or 8	Peripheral quantitative	No	Axial tension (4 weeks); axial compression (after 8 weeks)
Sigurdsen (2011) [25]	Tibia	40	Rats	No	4, 8.5	Micro	No	Bending test
Markel (1990) [17]	Tibia	32	Dogs	No	2,4,8, or 12	Quantitative	No	Torsional testing and indentation testing
Augat (1997) [20]	Tibia	28	Sheep	No	9	Peripheral quantitative	Yes	Nondestructive three-point bending test
Den Boer (1998) [28]	Tibia	24	Goats	Yes	2, 4, or 6	Axial spiral	No	Torsional testing
Wright (2012) [24]	Tibia	10	Mice	No	4	Micro	No	Torsion testing
Böhm (1999) [23]	Metatarsus	12	Sheep	No	8	Quantitative	No	Nondestructive three-point bending test

Linear relations between CT parameters and mechanical or histological outcomes were tested by performing Pearson’s correlation [21, 22, 25, 26, 29, 30], bivariate linear regression [17, 19, 20, 23, 24, 28], or multiple regression analysis [27, 29]. Böhm and Jungkunz (1999) also performed bivariate quadratic regression analysis [23].

### Parameters Generated with CT Representing Bone Union

#### Quantitative CT Parameters

Quantitative CT parameters that represent bone union are for example bone mineral density (BMD) and total volume of the callus (TV). Studies created volumes of interests (VOI) around the fracture, in which quantitative CT parameters were assessed. Table 5 shows the volumes of interests, bone thresholds, and outcome measures that were reported from CT. Also, it shows the parameters assessed from mechanical and histological testing.

**Table 5:** Outcome measures on axial CT cross-sections

Study	Volumes of interest	Voxel size (μm)	Energy settings (kV, mA)	Threshold for bone (mgHA/cm^3^)	Mechanical or histological testing outcomes	CT-outcomes
Mehta (2013) [29]	Fracture callus (region between the outer boundary of the callus and the periosteal surface of the cortex)	10.5	70, 114 mA	190	Torsional stiffness, peak torque	BMD, TMD, TV, BV, BV/TV, Tb.Th, Tb.N, Tb.Sp, σ_Tb.Th_, σ_Tb.Sp_, Da, Conn.D, SMI,
Morgan (2009) [27]	Area between the outer boundary of the callus and the periosteal surface of the pre-existing cortical bone, located between the proximal and distal boundaries of the callus	12	70, 114 mA	641.9	Torsional strength, angular deformation	TMD, BMC, TV, BV, BV/TV, σ_TMD_, J
Nyman (2009) [26]	Trajectory following the outer contours of the tissue; 3.2 mm above and below fracture line	32	55 and 145 mA	485	Maximum force, bending stiffness, energy to failure	BMD, BMD_Bridging Cortices,_ TV, BV, BV/TV, BV_Bridging Cortices_ /TV_Bridging Cortices_, Min BA/TA; Min BA_Bridging Cortices_ /TA_Bridging Cortices_, Scaled BV/TV, I
	Thin region of the outer cortices of first region of interest (representing bridging of the cortices)	32				
Shefelbine (2005) [30]	6.2 mm region around the fracture callus	34	NR	Semiautomatically thresholding to highly mineralized bone, newly mineralized bone, and soft tissue	Torsional rigidity	BMD_min_, BV, CSA (min and max), J, GJ, BJ (min, max, and mean)
Nazarian (2010) [19]	Osteotomy site and the adjacent bone	34	55, 145 μA	Assessed with global thresholding procedure	Torsional rigidity, peak torque, polar moment of inertia	GJ (min and mean)
Fiset (2018) [22]	1.8 mm length of diaphysis consisting of 250 slices that extend proximally from the most distal slice with disruption of the cortical ring of the femur	8	50, 800**μ**A	786	Torsional stiffness, peak torque, failure angle	BMD, TV, BV, BV/TV
Jämsä (2000) [21]	Three consecutive cross-sections with a slice distance of 1 mm. The central slice was at mid-callus	148	NR	0.5 cm^−1^ (total bone) 0.93 cm^−1^ (compact bone)	Failure load_tension_, Failure load_Compression_	BMD, BMD_CompactBone_, BMC, BMC_CompactBone_, CSA, CSA_CompactBone_
Sigurdsen (2011) [25]	Narrow region near fracture site and wide region encompassing more of the fracture callus region	50.7	NR	Soft callus: 171–540 Hard callus: 540–1200 Cortical bone: > 1200	Bending strength	BMD, TV, BV
Markel (1990) [17]	Four regions of interest (periosteal callus, endosteal callus, cortex, osteotomy gap)	NR	NR	NR	Peak torque, torsional stiffness, Angular deformation at failure, Indentation stiffness	BMD_osteotomy gap_, BMD_periosteal callus_, BMD_cortex_, BMD_endosteal callus_
Augat (1997) [20]	Seven consecutive transverse sections of which the central slice was at the plane of the former osteotomy	295	47, 0.3 mA	200 mg/cm^3^	Flexural rigidity	BMD; BMC; CSA
Den Boer (1998) [28]	Callus	NR	120, 165 mA	NR	Torsional strength, torsional stiffness	CD, CM
Wright (2012) [24]	Callus and fracture surface	8	50, 160μA	200 and 590	Peak torque, torsional stiffness	BMD, TMD, TV, BV, BV/TV, Tb.Th, Tb.N, Tb.Sp, SA, BA/TA, GJ (min., mean and surface)
Böhm (1999) [23]	Fracture gap automatically detected by algorithm	295	NR	NR	Bending stiffness, deformation	BMD, CD, BMC, J

*BMD* bone mineral density, *TMD* tissue mineral density, *TV* total callus volume, *BV* mineralized callus volume, *BV/TV* mineralized fraction of the callus, *BMC* bone mineral content, *CD* callus density, *CM* callus mass, *CSA* cross-sectional area, *GJ* torsional rigidity, *J* polar moment or inertia, *I* moment of inertia, *BJ* bending rigidity, *Da* degree of anisotropy, *Conn.D* connectivity density, *SMI* structure modeling index, *Tb,N* strut number, *Tb.Th* strut thickness, *Tb.Sp* strut separation, *SA* failure surface area, *BA/TA* minimal bone area per total area, *SSI* strength–strain index, *NR* not reported

#### Biomechanical CT Parameters

Three studies calculated the polar moment of inertia from CT [23, 27, 30]. Polar moment of inertia represents the resistance of bone to torsion and is dependent on the shape of the callus relative to the torsion axis. Polar moment of inertia is expressed as m^4^.

Three studies calculated torsional rigidity (GJ) of the fracture, based on CT-derived data [19, 24, 30]. GJ is a measure describing resistance of a bone when it is subjected to torsional forces and is expressed as Nm^2^. GJ is calculated from the cross-sectional area and CT-assessed bone mineral density. GJ was presented as an average of the entire VOI (GJ_AVG_) [19, 24, 30] and as the weakest slice of the VOI (GJ_MIN_) [19, 24]. Shefelbine et al. (2005) [30] also calculated the average bending rigidity.

### Associations Between CT and Mechanical or Histological Testing

#### Quantitative CT Outcomes with Mechanical Testing

The included studies used several quantitative parameters assessed from CT to represent bone union. The results of the studies are shown in Tables 6 and 7.

**Table 6:** Coefficients of determination for linear associations between CT-outcome measures and mechanical or histological testing outcomes

	Associations with mechanical or histological testing		
Bone mineral density (BMD)			
Mehta (2013) [29]	.04^B^	.04^D^	
Nyman (2009) [26]			
Bridging cortices	.05^G^	.05^H^	< .01^I^
Overall	.20^G^	.11^H^	.03^I^
Shefelbine (2005) [30]			
Minimum	.08^J^		
Fiset (2018) [22]	.04^B^	.22^D^	.19^E^
Jämsä (2000) [21]			
Compact bone	.09^A1^	.18^A2^	
Overall	.04^A1^	.32^A2^	
Sigurdsen (2011) [25]			
30 days post-fracture			
External fixator	< .01^F^		
Intramedullary nail	*.68*^*F*^		
60 days post-fracture			
External fixator	.25^F^		
Intramedullary nail	.01^F^		
Markel (1990) [17]			
Fracture gap	**.74**^**B**^	*.60*^*D*^	*.56*^*K*^
Periosteal callus	**.73**^**B**^	*.49*^*D*^	*.64*^*K*^
Cortex	n.s.^B^	n.s.^D^	n.s.^K^
Endeosteal callus	n.s.^B^	.35^D^	*.40*^*K*^
Augat (1997) [20]			
Minimum	**.70**^**L**^	*.62*^*M*^	**.71**^**N**^
Wright (2012) [24]			
0.2 gHa/CM3	n.s.^B^	n.s.^D^	
0.59 gHa/CM3	n.s.^B^	n.s.^D^	
Böhm (1999) [23]			
Hard bone	**.70**^**H**^		
Overall	**.75**^**H**^		
Callus density (CD)			
Den Boer (1998) [28]	**.82**^**B**^	**.72**^**C**^	
Böhm (1999) [23]	**.84**^**H**^		
Tissue mineral density (TMD)			
Mehta (2013) [29]	.31^B^	.28^D^	
Wright (2012) [24]			
0.20 gHa/CM^3^	*.60*^*B*^	n.s.^D^	
0.59 gHa/CM^3^	*.63*^*B*^	n.s.^D^	
Bone mineral content (BMC)			
Jämsä (2000) [21]			
Compact bone	.01^A1^		
	.32^A2^		
Overall	> .01^A1^		
	.20^A2^		
Augat (1997) [20]	.29^L^		
Böhm (1999) [23]	**.90**^**H**^		
Total callus volume (TV)			
Mehta (2013) [29]	.07^B^	.08^D^	
Nyman 2009	.07^G^	.18^H^	.05^I^
Fiset (2018) [22]	*.56*^*B*^	*.45*^*D*^	.30^E^
Sigurdsen (2011) [25]			
0.17–0.54 gHa/CM^3^; 30 days post-fracture			
External fixator	.21^F^		
Intramedullary nail	.26^F^		
0.17–0.54 gHa/CM^3^; 60 days post-fracture			
External fixator	.10^F^		
Intramedullary nail	.18^F^		
0.54–1.2 gHa/CM^3^; 30 days post-fracture			
External fixator	.23^F^		
Intramedullary nail	.12^F^		
0.54–1.2 gHa/CM^3^; 60 days post-fracture			
External fixator	.01^F^		
Intramedullary nail	.01^F^		
Wright (2012) [24]			
0.20 gHa/CM^3^	*.54*^*B*^	n.s.^D^	
0.59 gHa/CM^3^	*.54*^*B*^	n.s.^D^	
Mineralized callus volume (BV)			
Mehta (2013) [29]	.08^B^	.14^D^	
Nyman 2009	.10^G^	.07^H^	< .01^I^
Shefelbine (2005) [30]	< .01^J^		
Fiset (2018) [22]	*.60*^*B*^	*.67*^*D*^	*.47*^*E*^
Sigurdsen (2011) [25]			
30 days post-fracture			
External fixator	.17^F^		
Intramedullary nail	*.67*^*F*^		
60 days post-fracture			
External fixator	.15^F^		
Intramedullary nail	.19^F^		
Wright (2012) [24]			
0.20 gHa/CM^3^	**.80**^**B**^	*.55*^*D*^	
0.59 gHa/CM^3^	**.79**^**B**^	*.60*^*D*^	
Mineralized fraction of the callus (BV/TV)			
Mehta (2013) [29]	.01^B^	.01^4^	
Nyman 2009			
Bridging cortices	.20^G^	.17^H^	< .01^I^
Overall	< .01^G^	.03^H^	.12^I^
Fiset (2018) [22]	.27^B^	*.50*^*D*^	.38^E^
Wright (2012) [24]			
0.20 gHa/CM^3^	n.s.^B^	n.s.^D^	
0.59 gHa/CM^3^	n.s.^B^	n.s.^D^	
Cross-sectional area (CSA)			
Shefelbine (2005) [30]			
Minimum	< .01^J^		
Maximum	.01^J^		
Jämsä (2000) [21]			
Compact bone	.00^A1^		
	.16^A2^		
Overall	< .01^A1^		
	.04^A2^		
Augat (1997) [20]	.15^L^		
Callus mass (CM)			
Den Boer (1998) [28]	.05^B^	.02^C^	
Trabecular thickness (Tb.Th)			
Mehta (2013) [29]	.34^B^	.32^D^	
Wright (2012) [24]			
0.20 gHa/CM^3^	n.s.^B^	n.s.^D^	
0.59 gHa/CM^3^	*.63*^*B*^	*.52*^*D*^	
Trabecular number (Tb.N)			
Mehta (2013) [29]	.01^B^	< .01^D^	
Wright (2012) [24]			
0.20 gHa/CM^3^	n.s.^B^	n.s.^D^	
0.59 gHa/CM^3^	n.s.^B^	n.s.^D^	
Trabecular separation (Tb.Sp)			
Mehta (2013) [29]	.02^B^	.05^D^	
Wright (2012) [24]			
0.20 gHa/CM^3^	n.s.^B^	n.s.^D^	
0.59 gHa/CM^3^	n.s.^B^	n.s.^D^	
Standard deviation (Tb.Th)			
Mehta (2013) [29]	.31^B^	.31^D^	
Standard deviation (Tb.Sp)			
Mehta (2013) [29]	.14^B^	.18^D^	
Failure surface area (SA)			
Wright (2012) [24]			
Bone 0.2 gHa/CM^3^	**.73**^**B**^	n.s.^D^	
Bone 0.59 gHa/CM^3^	*.62*^*B*^	*.59*^*D*^	
Total 0.2 gHa/CM^3^	n.s.^B^	n.s.^D^	
Total 0.59 gHa/CM^3^	n.s.^B^	n.s.^D^	
Bone area per total area (BA/TA)			
Nyman 2009			
Bridging cortices	.29^G^	.21^H^	< .01^I^
Overall	< .01^G^	.06^H^	.05^I^
Wright (2012) [24]			
0.20 gHa/CM^3^	n.s.^B^	n.s.^D^	
0.59 gHa/CM^3^	n.s.^B^	n.s.^D^	
Degree of anisotropy (DA)			
Mehta (2013) [29]	.02^B^	.04^D^	
Connectivity density (Conn.D)			
Mehta 2013	.25^B^	.15^D^	
Structure modeling index (SMI)			
Mehta (2013) [29]	.10^B^	.12^D^	
Polar moment of inertia			
Nyman 2009			
Overall			
Mean	. 08^G^	.10^H^	.00^I^
Min	< .01^G^	.03^H^	< .01^I^
Bridging cortices			
Mean	< .02^G^	.09^H^	.07^I^
Min	< .01^G^	.01^H^	.09^9^
Shefelbine (2005) [30]	.04^J^		
Böhm (1999) [23]			
Calculated from center of mass	*.68*^*J*^		
Calculated from geometric midpoint	*.69*^*J*^		
CT-assessed torsional rigidity			
Shefelbine (2005) [30]	*.48*^*J*^		
Nazarian 2010			
Smallest	**.78**^**D**^	**.81**^**J**^	
Average	n.s.^D^	*.63*^*J*^	
Wright (2012) [24]			
Smallest	n.s. ^B^	n.s.^D^	
Average	*.50*^*B*^	n.s.^D^	
Surface	*.66*^*B*^	n.s.^D^	
CT-assessed bending rigidity			
Shefelbine (2005) [30]			
Smallest	*.49*^*J*^		
Largest	*.52*^*J*^		
Mean	*.52*^*J*^		

Bold indicate strong (*R*^2^ > 0.7) associations; italic indicate moderate associations (*R*^2^ 0.4–0.7); *A*1 failure load tension; *A*2: failure load compression; *B* torsional stiffness; *C* torsional strength; *D* peak torque; *E* failure angle; *F* bending strength; *G* maximum force; *H* Bending stiffness; *I* Energy to failure; *J* Torsional rigidity; *K* Indentation stiffness; *L* Flexural rigidity; *M* histologically % bone in periosteal callus; *N* histologically % bone in fracture gap; *T* coefficient of determination for failure load under tension; *C* coefficient of determination for failure load under compression; *n.s.* not significant, no coefficient of determination reported

**Table 7:** Coefficients of determination for quadratic associations between CT-outcome measures and mechanical or histological testing outcomes

	Outcome measures of mechanical or histological testing
Bone mineral density (BMD)	
Böhm (1999) [23]	
Hard bone	**.74**^**H**^
Overall	**.76**^**H**^
Callus density (CD)	
Böhm (1999) [23]	**.85**^**H**^
Bone mineral content (BMC)	
Böhm (1999) [23]	**.93**^**H**^
Polar moment of inertia	
Böhm (1999) [23]	
Calculated from center of mass	*.68*^*H*^
Calculated from geometric midpoint	*.69*^*H*^

Bold indicate strong (R^2^ > 0.7) associations; italic indicate moderate associations (R^2^ 0.4–0.7) H: Bending stiffness

Ten studies correlated bone mineral density (BMD) to mechanical outcome. Six studies did not find associations with R^2^ > 0.40 between BMD and mechanical outcomes [21, 22, 24, 26, 29, 30]. Four studies found moderate to strong associations with BMD [17, 20, 23, 25]. Böhm and Jungkunz (1999) also found strong associations for a quadratic association between BMD and mechanical testing [23].

Callus density (CD) was assessed by two studies, which both reported strong associations between CD and mechanical testing [23, 28].

Tissue mineral density (TMD) was assessed by two studies [24, 29]_._ One study reported weak associations [29], whereas the other study found moderate associations between TMD and mechanical testing [24].

For bone mineral content (BMC), two studies did not find associations with R^2^ > 0.40 [20, 21]. One study reported a strong linear and quadratic association for BMC with mechanical testing [23].

Total callus volume (TV) was assessed by five studies. Three studies reported no or weak associations between TV and mechanical outcomes [25, 26, 29]. Two studies reported moderate associations with mechanical outcomes [22, 24].

Mineralized callus volume (BV) was assessed by six studies. Three studies reported no or weak associations for BV with mechanical outcomes [26, 29, 30]. Three studies reported moderate to strong associations between BV and mechanical outcomes [22, 24, 25].

The mineralized fraction of the callus (BV/TV) was assessed by four studies [22, 24, 26, 29], of which one study found a moderate association [26].

Cross-sectional area (CSA) was assessed by three studies and was not associated with mechanical outcomes [20, 21, 30].

Some studies investigated less common CT-outcome parameters [24, 26–29]. From these parameters, associations with mechanical outcomes with *R*^2^ > 0.50 were found for trabecular thickness [24] and amount of bone across the failure surface area [24].

Morgan et al. (2009) and Mehta, Heyland, Toben, and Duda (2013) created regression models to associate mechanical outcomes to quantitative CT parameters [27, 29]. For maximum torque, a model with TMD, BMC, and σTMD explained 62% of the variation (*R*^2^ = 0.62), and a model with TMD, BV, and σTMD explained 61% (*R*^2^ = 0.61) [27]. For torsional rigidity, a model with TMD, BMC, BV/TV, and σTMD explained 70% of the variation (R^2^ = 0.70) [27]. Torsional stiffness could be predicted with a model containing strut thickness, the standard deviation of the strut separation, and strut number (*R*^2^ = 0.55). Torsional strength could be predicted with BMD or BV/TV, strut thickness, standard deviation, or strut separation (*R*^2^ = 0.57).

#### Quantitative CT Outcomes with Histological Testing

Augat et al. (1997) was the only study who correlated CT-outcomes to histological outcomes. They reported a moderate association (*R*^2^ = 0.62) between minimal BMD and histologically assessed percentage bone in periosteal callus. A strong association (*R*^2^ = 0.71) was reported between the minimal BMD and histologically assessed percentage bone in fracture gap.

#### Biomechanical CT Outcomes with Mechanical Testing

Polar moment of inertia was assessed by three studies. Two studies found no or weak associations between moment of inertia and mechanical outcome [26, 30]. Böhm and Jungkunz (1999) reported moderate linear and quadratic associations between polar moment of inertia and mechanical testing [23].

Three studies associated CT-assessed torsional rigidity to torsional rigidity assessed by mechanical testing [19, 24, 30]. All three studies reported moderate to strong associations between the average torsional rigidity and mechanical testing results [19, 24, 30].

Shefelbine et al. (2005) reported moderate associations between CT-assessed maximum and mean bending rigidity and mechanical outcomes [30].

### Data Synthesis

Overall, for two parameters, all studies investigating these parameters found moderate or strong associations. These parameters were CD, which was assessed by two studies, and CT-assessed torsional rigidity, which was assessed by three studies. For BMD, TMD, BMC, TV, BV, trabecular thickness, and polar moment of inertia, 30–60% of the studies investigating these parameters found associations. For BV/TV, CSA, trabecular number, trabecular separation, and bone area per total area, less than 30% of the studies found an association for these parameters.

Some parameters were only assessed by one study. From those, CT-assessed bending rigidity and amount of bone across the failure surface area showed moderate to strong associations.

## Discussion

We aimed to identify CT-outcome parameters which are associated most strongly with bone union after a fracture. The associations found by the studies are conflicting, with exception for CT-assessed torsional and bending rigidity, and callus density.

CT-assessed torsional rigidity was found to have moderate to strong associations by all three studies that investigated it. Torsional rigidity is calculated from CT-acquired data and is dependent on the callus density, cross-sectional area, and the distribution of bone density within the callus [19, 30]. Based on CT, virtual models of the bone are created on which virtual mechanical testing can be performed. From this virtual testing, torsional rigidity is calculated [19, 30, 31]. Average torsional rigidity showed moderate associations with mechanical tests in all three studies [19, 24, 30]. The results of Naziarian et al. (2010) [19] showed that minimum torsional rigidity had a stronger association with mechanical testing than average torsional rigidity. This means that analyzing only the weakest segment (axial slice) of CT images would give the strongest associations. This seems logical, as failure of a beam under forces is dependent on the weakest point, and not the average strength [19]. However, Wright et al. (2012) [24] did not find an association between minimum torsional rigidity and mechanical testing. According to Wright, the use of the tibia, and not the femur as Nazarian did, might explain this [24]. In contrast to the femur, the diameter of the tibia decreases when going more distally. As torsional rigidity is dependent on the CSA, the minimum torsional rigidity might therefore move to the most distal part of the VOI when analyzing the tibia [24]. This once more indicates that the assessment of fracture healing is complex and dependent on many variables.

This complexity may have led to the conflicting results of the other parameters. For example, quite strong association were reported for BMD by three studies, whereas other studies found no associations with BMD. Because of the conflicting results between studies, the generalizability of the associations seems to be quite low. Also, most studies in this review explored linear relations, but Böhm and Jungkunz (1999) showed that associations might be quadratic [23]. However, Böhm and Jungkunz (1999) was the only study investigating quadratic associations and it was a small study (*n* = 12).

So far, CT-assessed torsional rigidity seems a promising parameter for bone union assessment. Clinically, several studies have been investigating CT-assessed torsional rigidity. CT-assessed torsional rigidity was successfully used for the prediction of fractures in patients with bone lesions [32–34]. Also, recently, the first clinical study has been published that used CT-assessed torsional rigidity to assess tibial fracture healing [35]. In this study, a low-dose CT was made of the tibia 12 weeks after surgical fixation. Software was used to create a virtual model of the fractured tibia which was adapted to a model of an intact tibia. Virtual torsional testing could then be performed on these models, resulting in torsional rigidity values for the fractured and intact tibia. Lastly, torsional rigidity of the fractured model was divided by the torsional rigidity of the intact model. By doing this, a dimensionless parameter was created which indicates the progression of healing relative to the intact tibia [31]. Given the results of this review, and the promising results of the first clinical study, CT-assessed torsional rigidity could become a useful tool for bone union assessment. However, at this moment, the clinical applicability of CT-assessed torsional rigidity is limited. Advanced software and knowledge are needed to conduct CT-based structural rigidity analysis (CTRA) [32]. Although CTRA can be done with data from any CT-scanner, bone densities are very important for the analysis. Therefore, phantoms with known bone densities should be scanned with the patient [32].

This systematic review encountered some limitations. Firstly, CT-assessed torsional rigidity and callus density were only assessed by a limited number of studies (three studies for CT-assessed torsional rigidity and two studies for callus density). Although those studies show promising results, more studies should be done to further confirm these results. Parameters that were assessed by more than three studies had higher chances of finding contradictory results. However, the more investigated parameters in this review showed no significant associations in most of the studies. BMD for instance was investigated by ten studies, of which only four reported significant associations. A second limitation of this review is that the statistical associations that are presented come from animal studies. We should be careful by translating these results directly to clinical human fractures, as data retrieved from animal studies might be unreliable in clinical studies [10]. For example, studies have shown that rodent bone remodeling is different from large animal or human bone remodeling because it is lacking intracortical remodeling [36]. Therefore, associations for bone healing might be different for rodents compared to large animals or humans. Also, most studies in this review used micro CT-scanners with higher spatial resolutions and higher radiation doses than clinical CT-scanners [19, 21, 26, 27]. Therefore, clinical CT-scanners might be less accurate than micro CT-scanners [37]. Thirdly, for this systematic review, we used fairly strict inclusion criteria. The main reason for these strict criteria was to keep heterogeneity between studies as low as possible to be able to compare studies and therewith draw a firm conclusion. Even with these strict criteria, the heterogeneity between studies was high. Studies used different location of fractures, animal species, scanning protocols, and mechanical testing protocols, which is likely to affect the associations found between the studies. Also, four studies used drug treatments to increase fracture healing [19, 26–28]. These treatments can modulate structural and mechanical properties of the callus [38]. Due to the strict inclusion criteria, many studies were excluded during the study selection process. These were also studies who assessed bone healing by performing CT, mechanical and histological testing. However, in these studies, the different methods were used complementary to each other and the results of these methods were not compared to each other. Therefore, it is not possible to draw conclusions from these studies concerning the best CT-outcome parameter. Also, minimal follow-up time was set to 4 weeks, as we were not interested in studies who only looked at early stages of fracture healing. As fracture healing progresses differently between animal species and depends on fracture size, one could argue if this period was accurate. Also, the associations between CT parameters and mechanical and histological outcomes might be influenced by the stage of fracture healing, which may vary between the studies. Lastly, the risk of bias of studies was assessed with the QUADAS-2 tool. As this tool is designed for clinical studies, it may not be accurate for pre-clinical studies. However, no pre-clinical risk of bias tool exists for diagnostic studies. Most studies in this review showed concerns about risk of bias. To decrease risk of bias in future studies, we strongly recommend to interpret the index test (CT), without knowing the results of the reference test (mechanical or histological testing) and to describe this process in the paper.

Based on the currently available literature, density-related parameters seem to be most promising parameters to assess bone union after a fracture. Especially, CT-assessed torsional rigidity is a promising parameter to assess bone union. To improve the clinical assessment of fracture healing, we encourage the conduction of more high-quality clinical studies investigating the applicability of CT-assessed torsional rigidity for bone union assessment. In the future, torsional rigidity could potentially become a widely accepted outcome measure for bone union assessment in clinical studies and in clinical practice.

## Data Availability

Data available within the article.
